# Rare Earths—The Answer to Everything

**DOI:** 10.3390/molecules29030688

**Published:** 2024-02-01

**Authors:** Thomas Behrsing, Victoria L. Blair, Florian Jaroschik, Glen B. Deacon, Peter C. Junk

**Affiliations:** 1 School of Chemistry, Monash University, Melbourne, VIC 3800, Australia; thomas.behrsing@monash.edu (T.B.); victoria.blair@monash.edu (V.L.B.); glen.deacon@monash.edu (G.B.D.); 2 ICGM, Univ Montpellier, CNRS, ENSCM, 34090 Montpellier, France; florian.jaroschik@enscm.fr; 3 College of Science & Engineering, James Cook University, Townsville, QLD 4811, Australia

**Keywords:** rare earths, sustainability, environment, recycling, corrosion inhibitors, agriculture, catalysis, health, materials

## Abstract

Rare earths, scandium, yttrium, and the fifteen lanthanoids from lanthanum to lutetium, are classified as critical metals because of their ubiquity in daily life. They are present in magnets in cars, especially electric cars; green electricity generating systems and computers; in steel manufacturing; in glass and light emission materials especially for safety lighting and lasers; in exhaust emission catalysts and supports; catalysts in artificial rubber production; in agriculture and animal husbandry; in health and especially cancer diagnosis and treatment; and in a variety of materials and electronic products essential to modern living. They have the potential to replace toxic chromates for corrosion inhibition, in magnetic refrigeration, a variety of new materials, and their role in agriculture may expand. This review examines their role in sustainability, the environment, recycling, corrosion inhibition, crop production, animal feedstocks, catalysis, health, and materials, as well as considering future uses.

## 1. Introduction

In this review essay, we will see how well the claim in the title stands up. It can be examined in the light of major current issues such as sustainability, the environment, transport, food security, and health. The rare earth elements now play critical roles in many areas of modern technology including magnets (neodymium and dysprosium) in wind turbines, automobiles, computers, and military guidance systems; lighting phosphors (europium and terbium); exhaust emission catalyst support ceramics; and industrial catalysts (lanthanum, cerium, and neodymium) [1,2,3,4]. Most people in developed economies encounter them every day without realizing it. Rare earths are regarded as critical materials. This account is a critical opinion piece rather than a comprehensive review.

**Figure 1:**
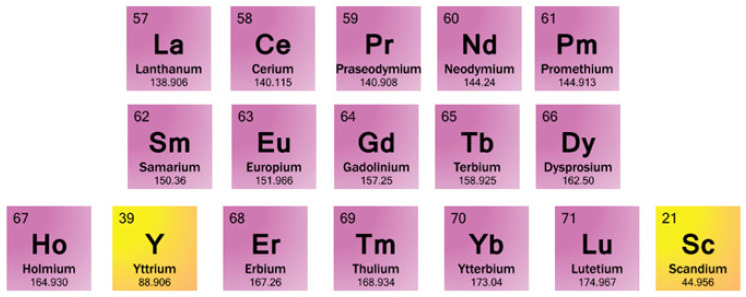
Rare earth elements ordered in decreasing trivalent ion size from lanthanum to scandium.

Rare earths are the group 3 metals, scandium and yttrium, together with the lanthanoids, La-Lu inclusive (Figure 1). Use of the term rare earths requires that at least one of either scandium or yttrium is involved. “Lanthanoid” is the IUPAC term for elements in the La-Lu group. The frequently used “lanthanide” is non-IUPAC and carries implications of an anionic species; hence, its use should be phased out. At the recent inaugural Australian rare earth chemistry meeting (OZRE23) of the Royal Australian Chemical Institute, it was noteworthy that most participants used “lanthanoid”.

This review is being considered within the framework of “Inorganic Chemistry are we relevant?” With respect to rare earths, the answer is a resounding “Yes”!

## 2. Discussion

With regard to sustainability, there are at least two issues to be considered here. Firstly, how can rare earths contribute to sustainability in terms of energy generation and transport? Secondly, how sustainable is the supply and use of rare earths?

### 2.1. Energy Generation and Transport

**Figure 2:**
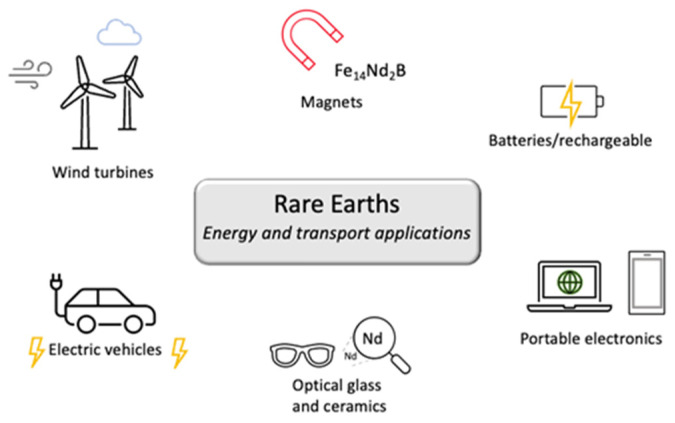
Selection of energy and transportation uses of rare earth metals.

Rare earths play a key role in green energy generation (Figure 2), as the rare earth magnet, Fe_14_Nd_2_B, the strongest known permanent magnet, is a vital part of wind generators for clean energy production. In addition, magnets are used in all cars but especially so in hybrid and electric cars, where kilogram amounts are used [1,5]. These uses will contribute to a cleaner, lower carbon environment. The magnets are also used in computers and mobile phones, without which it is hard to see us progressing. The magnets also contain the less abundant Dy and may also have Pr to inhibit corrosion. Ceria-based ceramics are the supports for exhaust emission catalysts which lead to a cleaner environment, representing the major use of rare earths in the USA in terms of amount [4]. They also have an active role in the catalytic removal of greenhouse gases [6]. It is thus optimal that cerium is ”he m’st abundant rare earth. However, this use will gradually disappear as petrol- and diesel-powered vehicles are phased out (and there is thus scope for investigating new applications for cerium).

### 2.2. Supply, Processing, and Separation

#### 2.2.1. Supply

**Figure 3:**
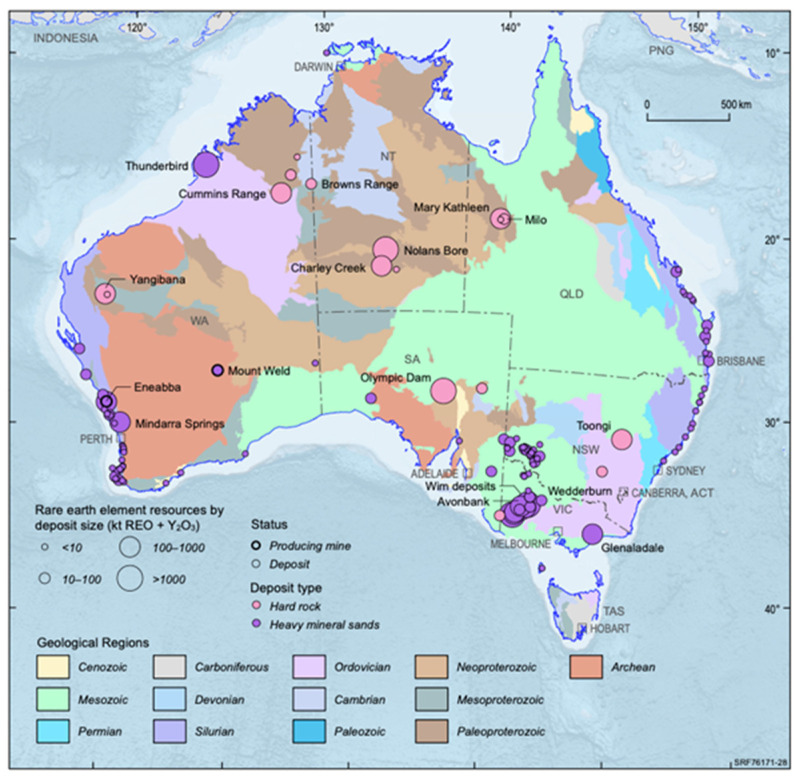
Australian rare earth element deposits and operating mines, 2021. For clarity, only major or significant deposits are labelled. Deposit size is based on total resources. Reproduced with permission from Geoscience Australia [7].

Rare earths are not rare. Cerium, the most abundant, is more abundant than tin, lead, or copper, none of which are viewed as rare. Thulium, the least abundant, is more prevalent than iodine. Apart from the major reserves in China and Mongolia, Australia has considerable reserves (Figure 3), which are regularly added to, both with new discoveries and the expansion of existing ore bodies. The Wimmera (Victoria) deposit is vast and will be a huge source once chemical engineering issues are solved. A new clay-based deposit in South Australia extends from Keith through Naracoorte to the Victorian border. Then, there are Nolans in the Northern Territory, now with significant investment from Hancock Prospecting, Australian Strategic Minerals in NSW, Hasting’s two deposits with investment from Wyloo Metals, and Northern Minerals have at least three deposits now linked with Iluka, to add to Lynas Rare Earths’ large Mt. Weld ore body in Western Australia. Uranium tailings dams are another potential source. Japan plans to extract rare earth-containing muds from deep sea deposits to reduce dependence on China. However, it is one thing to have resources; it is quite another to have separated rare earths and rare earth magnets.

The People’s Republic of China holds a significant portion (37%) of the world’s reserves of rare earths, but for the last two decades, it has supplied approximately 90% of the world’s rare earth oxides, which represents a perilous reliance [1]. Europe also holds a substantial amount of rare earth deposits [8], but mining start-up costs, environmental factors, and the threat of public disapproval and protests means that no rare earth mining occurs in Europe today [8]. However, a recent discovery of a large ore body in North Sweden could change this [9]. Very recently, Swedish iron ore miner LKAB announced it had discovered Europe’s largest known deposit of rare earth elements in Kiruna, situated in the province of Lapland in the far north of Sweden. It has been estimated that the deposit contains 1 million tonnes of rare earth oxides. If mined, this could enable a green transition for the EU’s self-sufficiency and independence from Russia and China, who could restrict REE exports for economic or military leverage. It is estimated, however, that it could take “10 to 15 years” before the materials enter the market [9].

With China dominating the supply of these—at one stage about 97%—the dangers of this situation were realised in 2010–2011, when China banned exports of rare earths to Japan over a territorial dispute. The shockwaves led to the reopening of the Mountain Pass Mine in the USA, the re-floating of Molycorp at USD 14 a share, rising at one stage to USD 74, a shake-up in ownership of rare earth companies, and then to the eventual bankruptcy of Molycorp with shares at USD 0.38. The fall-out from all of this has led to Lynas Rare Earths, an Australian company, supplying about 10% of the supply of separated rare earths, and they have the US Department of Defense’s support for both light and heavy rare earth separation plants in Texas [10]. Given the industrial importance of rare earths, an assured supply of processed rare earths has become essential [1,3,4,5].

**Figure 4:**
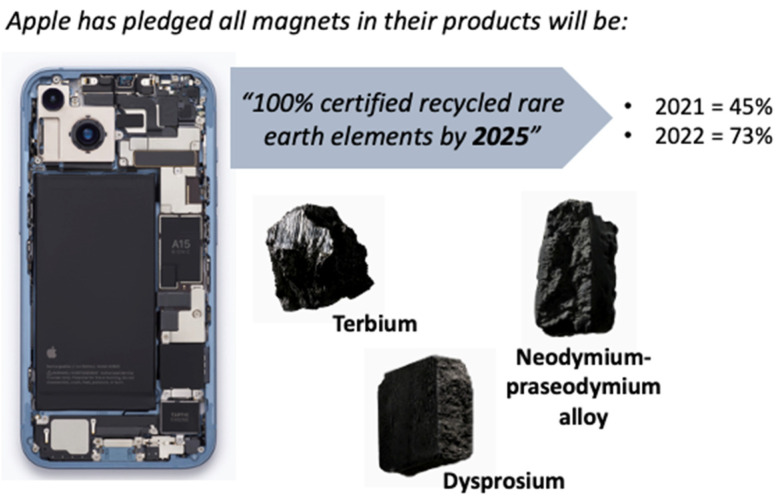
Apple’s^TM^ pledge to use 100% recyclable rare earth elements in all its products by 2025. Figure adapted from Apple Newsroom article [11,12].

There is considerable potential in recycling, particularly of rare earth magnets, and Apple has committed to using only recycled materials in their products (Figure 4). Although this area is in its infancy, considerable efforts are being made, and ionic liquids are being examined as part of the solution (see Section 2.3). There is an increasing demand for these elements and growing concerns over their secure supply [1,3] following their recent designation as Critical Raw Materials according to the European Commission, reflective of their projected future demand, economic availability, political influences, effectiveness in recycling, and potential difficulty for substitution. Recycling is discussed in a separate section below.

#### 2.2.2. Processing and Separation

The biggest challenges to sustainability and the environment currently lie in processing. The radioactivity associated with rare earth deposits (from U and Th) has historically caused problems such as slightly radioactive playgrounds in Eastern Australia from the misuse of tailings. Radioactive waste has caused problems for Lynas Rare Earths in Malaysia with such waste from earlier processing having to be returned to Australia, and the firm is now required to remove U and Th before exporting ore to the Malaysian plant. To this end, a concentration plant is being built in the WA goldfields region at Kalgoorlie [13]. It tends to be forgotten that radioactive material is not generated in rare earth processing. It was present in the original ore body. Thus, after it is removed from the concentrate and suitably diluted with soil, other waste minerals, etc., to the radiation level of the original ore body, it can be returned to the former location with no radiation change from the original environment.

The methods for the removal of thorium and uranium from rare earth ores prior to processing have been reviewed [14]. The emphasis is on the removal of thorium as it is far more prevalent in most ore bodies, which often have very low U levels. Major methods involve precipitation of Th as pyrophosphate or oxide/hydroxide. Thorium precipitates at pH values lower than those that cause significant *RE* precipitation. The foregoing review covers the removal of Th/U as practiced in the main Chinese plants, the method followed at Mountain Pass, the method used by Lynas in Malaysia, and prospective methods for some Australian ore bodies such as the Nolans. The development of the Arafura Resources Nolans ore body requires a different approach as it is a *RE*/U deposit and production of both uranium oxide and rare earths as well as phosphoric acid is planned [15].

**Figure 5:**
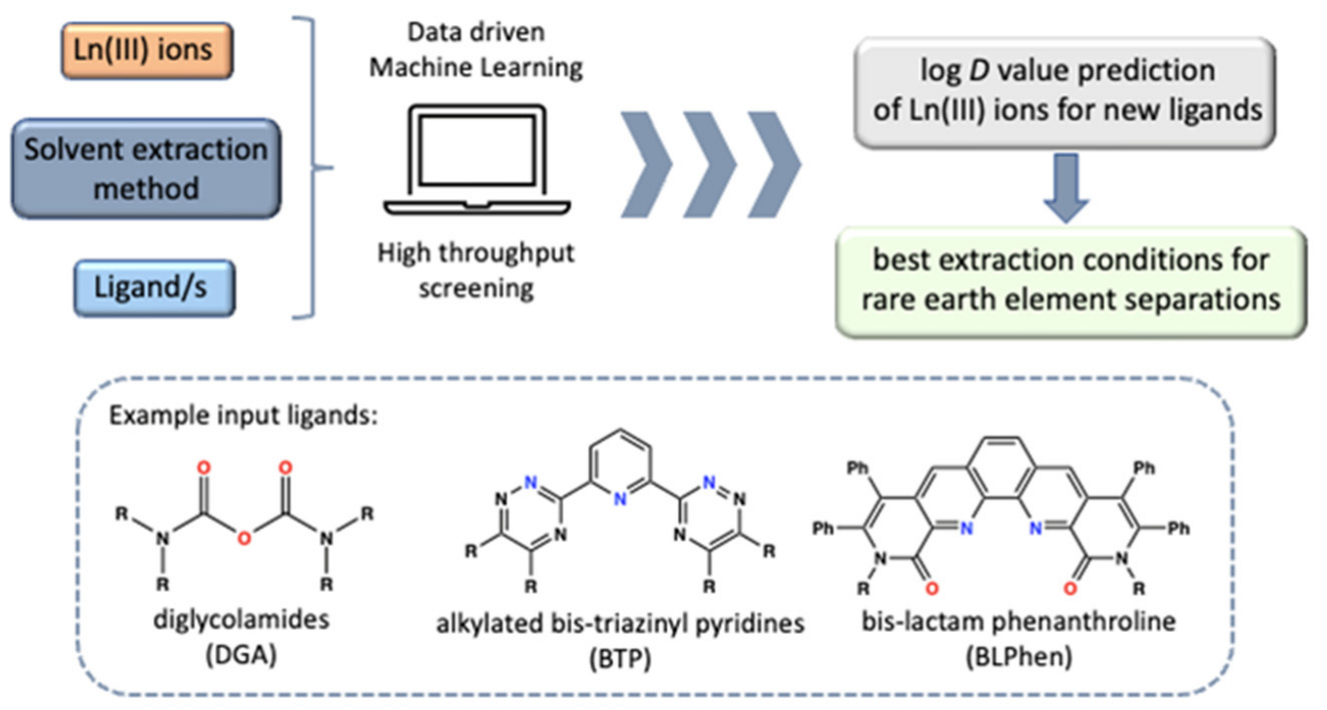
Machine learning approach to advance rare earth element separation [16].

The predominant use of solvent extraction for the separation of rare earth elements presents environmental issues, owing to the use of phosphoric acid esters and organophosphate extractants and of substantial volumes of kerosene. Recent reviews of separation methods [16,17] consider current procedures as well as some alternative technologies including the use of ionic liquids in extraction. Amine, amide, and carboxylic acid (e.g., naphthenic acid) extractants have better environmental credentials but are less effective in separation. Ion exchange column chromatography, which has been somewhat sidelined by the cost of absorbents and scaling issues, is more environmentally friendly and improvements have been made [16]. Traditional fractional crystallization, the initial separation method, is extremely tedious, but when carried out in water, it is environmentally optimal. Use of different salts or double salts may yet improve differentiation, and an effective Nd/Dy crystallization separation has been provided to enable the recovery of these elements from magnets [18]. Magnetically assisted crystallization has been reported [19] and has been used in rare earth separations [20]. There has been a recent report of the use of machine learning to advance rare earth separation (Figure 5) [21]. The authors showed that neural networks trained on available separation data and physicochemical ligand descriptors could be used to predict the behaviour of new ligands in solvent extraction separations. Computer predictive software was used to identify potential ligands for solvent extraction. In this approach, ligands in an organic phase are screened for high distribution ratios where *D* = [M^3+^]_org_/[M^3+^]_aq_. A high *D* value is a measure of a predicted formation of a stable Ln^III^ complex and a range of promising diglycolamides, alkylated bis-triazinyl pyridines, and 2,9-bis-lactam-1,10-phenanthrolines have been identified to date. These were synthesized and tested and showed good agreement with the predictions. The value of this approach to reduce trial and error experimentation is likely to enable the optimization of systems related to those already being studied rather than generating entirely new ligand systems. A high-throughput method has also been developed for screening the separation of rare earth elements, based on precipitation with pH changes [22].

**Figure 6:**
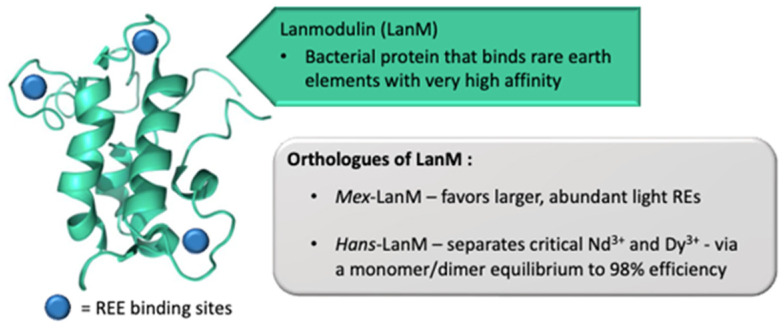
Lanmodulin protein structure (from Trenzyme GmBH) with its selective rare earth element (REE) binding sites highlighted.

Another approach under development includes the isolation of rare earth elements from highly dilute aqueous solutions through biosorption with microalgae, such as *Physcomitrella patens* (a primitive moss), with initial studies being conducted on neodymium and europium [23]. Even more exciting are developments involving the Lanmodulin protein, which has been shown to selectively remove rare earths from a mixture of potentially competing ions including Ca^2+^and Mg^2+^ [24]. This approach was then developed into a method for separating Nd^3+^ and Dy^3+^ with 98% efficiency (Figure 6) [25]. Biological and bio-inspired separation methods have been used to separate Ln^3+^ and An^3+^ [26]. Whilst these developments of new separation methods and modified methods are welcome, there is quite a step to having them adopted as industrial processes to reduce the environmental footprint of current separation methods. The biochemical separation methods [24,25] are a new paradigm but need to be shown to be scalable and economic.

### 2.3. Rare Earth Recycling and Recovery

#### 2.3.1. Introductory Comments

Both from a conservation viewpoint and the need for a circular economy, the recycling of rare earths is desirable. However, whilst there are abundant rare earth mineral reserves, limited processing capacity/competition, and a supply/demand balance, there is a limited incentive to recycle. As Binnemans [2] has pointed out, market intervention by way of incentives or legislative requirements is needed to promote the adoption of recycling at an industry level. Indeed, pilot plants or larger magnet reprocessing are currently being supported by government agencies such as the US Department of Defense [2]. However, current recycling is generally considered to account for <1% of total production [2]. Thus, end-of-life devices now need to become a strategic secondary source of these critical elements. Recovery from bulk uses such as Fe-Nd-B magnets, exhaust emission catalyst supports, and the vast amounts of spent fluid catalytic cracking (FCC) catalysts is clearly more attractive than more disseminated sources such as fly ash and electronic waste. Even with 1–5% of the FCC catalysts, the rare earths are more abundant than in the disseminated sources. To some extent, spent magnets and catalyst supports might be more attractive input sources for existing separation plants than primary ore. With the former, dissolution in the appropriate acid, selective precipitation of iron taking advantage of the basicity of Fe_2_O_3_ being less than that of RE_2_O_3_, would provide a solution suitable for normal solvent extraction separation. In the case of the latter, dissolution, pH adjustment, and oxidative precipitation of CeO_2_ would enable Ce reuse.

#### 2.3.2. Disseminated Sources

More disseminated sources such as electronic waste, fluorescent tubes’ fly ash, and waste water are less attractive, more expensive, and generally more challenging chemically, though all have advocates. Such end-of-life products can contain low levels of rare earths, so further work is required to enhance separation technology efficiencies to make these processes truly viable.

Although the first of these is often touted as a potential rare earth supply [27], an assessment by a series of panels as to priorities in the recovery of critical materials from waste electronic and electrical equipment ranked rare earth metals as of little importance from this source [28]. Even more noteworthy, a review on the recycling of metals from waste-printed circuit boards does not list rare earths as possible outputs [29]. Nevertheless, the pyrolysis of a range of printed circuit boards at 850 °C led to a concentration of rare earth elements in the carbonaceous residues (but not in metallic residues) sufficient to be utilised for urban mining [30,31]. A study of industrially pre-treated waste-printed circuit boards found that rare earths were concentrated in fine particulates in contrast to most other metals [27]. The authors proposed a simple sieving step as a prelude to attempted recovery.

Whilst fly ash from burning coal for power generation contains significant levels of rare earths [32], there are problems in reclaiming them. Fly ash also contains a significant amount of toxic metals, e.g., Hg, Cd, Pb, and U, that would have to be removed and dealt with. A large amount of silica or other siliceous materials has to be separated. There are likely to be significant levels of alkaline earths, especially calcium, elements whose salts have a similar solubility profile to the trivalent rare earth salts. Furthermore, fly ash has two main types, C (high Ca) and F (lower Ca) [33], quite apart from compositional variations within the classes; hence, each supply presents unique problems for rare earth recovery. One size does not fit all.

#### 2.3.3. Concentrated Sources

Because Fe_14_Nd_2_B contains ca. 30% Nd by weight [2], spent magnets are attractive for recycling. They also contain 1–5% of the less abundant and more expensive dysprosium to enhance the magnetic properties. They may contain praseodymium for anti-corrosion purposes [34], and Pr may be substituted for Nd [2]. For these reasons, model systems for recycling/recovery from magnets usually focus on the separation of neodymium from dysprosium [35,36]. The recycling of magnet materials has recently been reviewed [34,37,38], as well as being included in an earlier wider review of the recycling of rare earths [39]. As magnets are used in a range of domestic and industrial electronic devices, e.g., motors and large-scale electricity generators such as modern wind turbines, large amounts of end-of-life magnets will become available. Several technologies have been developed including a process called hydrogen processing of magnet scrap (HPMS) to recover Nd–Fe–B alloy powders from end-of-life waste streams, and it is currently being scaled to test commercial viability [2].

**Figure 7:**
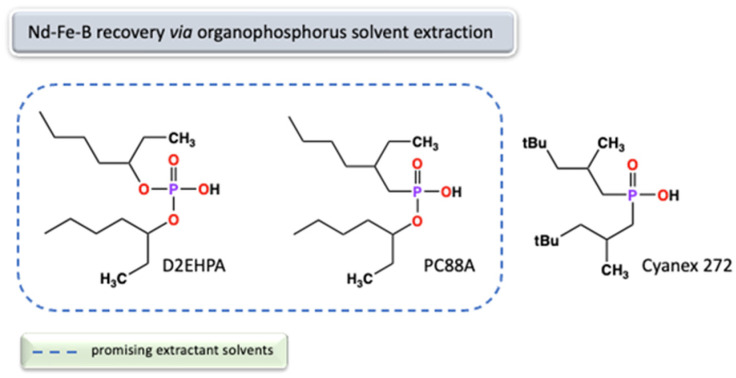
Organophosphorus/phosphate ester-based solvents for extraction and recovery of Nd-Fe-B alloys.

Another approach for Nd–Fe–B recovery is a solvent extraction route using phosphorus esters, such as the di(2-ethylhexyl) phosphoric acid called D2EHPA [40], or using saponified 2-ethyl-hexyl phosphonic mono-2-ethyl-hexyl ester (EHEPA or PC88A) and bis(2,4,4-trimethylpentyl)phosphinic acid (Cyanex 272) (Figure 7) [34]. A recent review by Yang and co-workers [38] identified that DEHPA and PC88A were the most effective and promising extractants. In both cases, flammable kerosene was preferably used as a diluent. Such processes replicate environmental challenges faced by primary rare earth separation.

**Figure 8:**
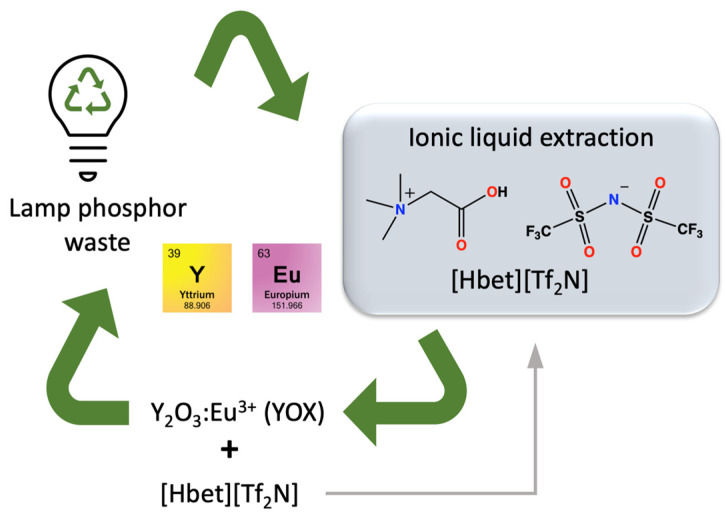
Ionic liquid extraction of Y and Eu from lamp phosphor waste.

Other work on Nd–Fe–B recovery has focussed on ionic liquids. Although these liquids are non-flammable alternatives to kerosene used in solvent extraction, several non-desirable characteristics of ionic liquids create issues. These include their higher viscosity because of their ionic composition. High viscosity reduces mass transport and so leads to losses of the ionic liquid by a tendency to extract via an unwanted ion exchange mechanism. Filtration difficulties, recyclability challenges, and higher prices are other issues. Dupont and Binnemans [41] have published a new recycling process for microwave-heated NdFeB magnets, using the carboxyl-functionalized ionic liquid betainium bis(trifluoromethylsulfonyl)imide, [Hbet][Tf_2_N]. In this, a leaching/extraction step traps Fe in the ionic liquid phase, while Nd, Dy, and Co are retained in the aqueous phase. Selective stripping and precipitation steps give a mixture of Nd_2_O_3_/Dy_2_O_3_ (separated further in an additional process) and cobalt as CoO. The ionic liquid [Hbet][Tf_2_N] can be regenerated for reuse. A system for the separation of Nd and Dy from magnets has also been reported by Schelter [22] using the tripodal nitroxide ligand [{(2-^t^BuNO)C_6_H_4_CH_2_}_3_N]^3-^ which is able to trap rare earth ions selectively. Binnemans has also used the [Hbet][tf_2_N] ionic liquid in a process for the recovery of Y_2_O_3_:Eu from lamp phosphor waste (Figure 8) [41], whilst this ionic liquid has also been used in a one-step separation of light from heavy rare earths [42]. Biological separation methods [24,25,26] may also develop a place in recycling. Recovery from FCC catalysts appears relatively underdeveloped [43], but it has considerable potential.

The need for the recycling of rare earths will continue to grow as new technologies emerge and recycling expectations become more important to consumers seeking sustainable manufacturing practices. 

### 2.4. Rare Earth Corrosion Inhibitors—A Contribution to Sustainability

One often less considered feature of sustainability is the maintenance of existing structures. This is usually discussed in terms of the preservation of historic buildings, landscapes, indigenous art in exposed settings, and historic urban streetscapes. However, the maintenance of major urban infrastructure, namely, bridges, commercial buildings, pipelines, factories, and transport infrastructure such as railways, planes, and ships, is an important area of sustainability. The great enemy of infrastructure sustainability is *corrosion*. It is a silent enemy. The study of corrosion and its mitigation is unfashionable and certainly not headline-grabbing. However, the failure of infrastructure owing to corrosion, bridges collapsing, ships sinking, pipelines rupturing, planes crashing, and trains derailing makes headlines.

Corrosion is a persistent, widespread, and costly problem. It costs the world around USD 4 trillion annually [44] and in the United States, according to a survey, it costs six cents for every dollar of gross domestic product [45]. Corrosion affects land and offshore constructions; auto, aviation, chemical plant, and water treatment facilities; water cooling towers; and the safety and durability of the huge network of underground infrastructure assets such as oil and gas pipelines. These sectors are vital for the provision of the world’s essential services and the maintenance of its economic activities. Corrosion can also lead to catastrophic failures in aircraft, bridges, and pipelines, as seen from pipeline explosions in China [44]. Understanding corrosion and developing and assessing methods of mitigation are fundamental scientific challenges with immense practical consequences [46]. Corrosion is a thermodynamic phenomenon whereby the metal reverts by oxidation to the most stable oxidation state.

**Figure 9:**
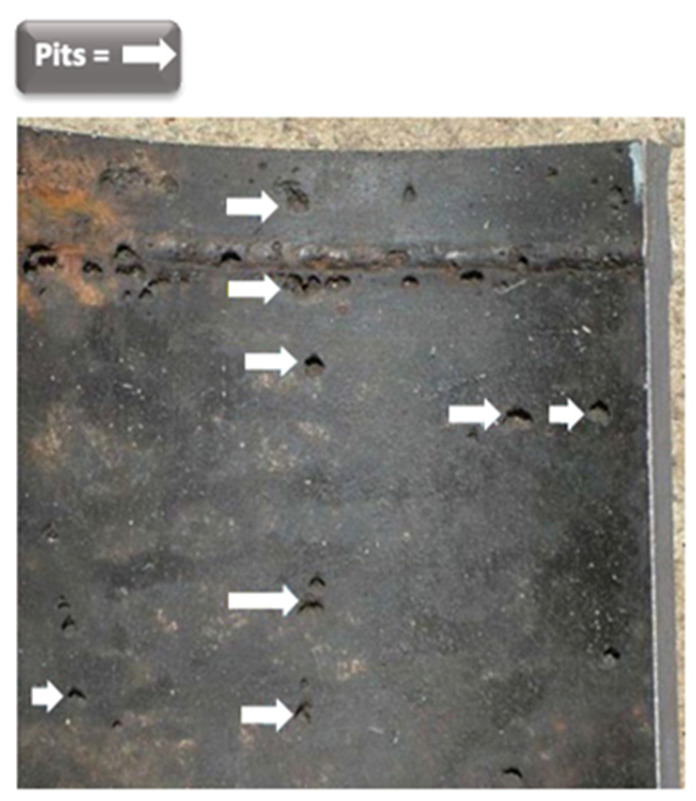
Pitting corrosion, photo of a section of an X65 steel pipe from an oil field in Western Australia.

A common way of preventing corrosion (e.g., pitting, Figure 9) is through the use of chemical inhibitors. This is particularly important for the protection of pipelines against internal corrosion, as cathodic protection only obviates external corrosion. Chromate(VI) salts have been excellent inhibitors for many metals for over 80 years [47], and they are used in pickling solutions, etchants, anodising and conversion coatings, primer paints, and sealants, but major pollution occurs in production areas [48]. The toxic nature of the chromate ion is well known [49], and for almost 30 years, research has focussed on finding an environmentally acceptable replacement. Rare earth salts have emerged as possible alternatives [50,51,52]. Thus, cerium chloride [53] was found to be an excellent inhibitor for aluminium alloys, and CeCl_3_ in flowing tap water reduces the corrosion rate on mild steel by a factor of 10 [54]. Their widespread use in agriculture in China [55] and their use as animal feedstocks [56] attest to their low toxicity and their environmental credentials.

Recent innovative developments have involved combining rare earths with organic corrosion inhibitors, notably carboxylates and phosphate esters, to produce bifunctional inhibitors [52,57]. Simple arenecarboxylate salts function as anodic inhibitors (but high concentrations are needed) and rare earth salts often are cathodic inhibitors; hence, some rare earth carboxylates provide an effective dual-function inhibition of corrosion of steel with synergism being possible [52,57]. Thus, it was found that cerium salicylate [57], lanthanum 4-hydroxycinnamate [58], and recently some rare earth 3-(4′-methylbenzoyl)propionates [59] are effective systems for mild steel [60,61,62,63]. Advances in the protection of aluminium-based aircraft materials have been achieved with cerium dibutyl phosphate [64], and a cerium organophosphate has been formed in situ and used as a quick repair agent for carbon steel [65]. However, organophosphates are environmentally less attractive than carboxylates. Further, rare earth cinnamates were shown to be excellent corrosion inhibitors for steel in chloride electrolytes saturated with CO_2_, conditions relevant to oil pipeline corrosion [66].

Before the widespread adoption of rare earth corrosion inhibitors takes place, some issues have to be resolved. The choice of the rare earth element is unclear as different carboxylates perform best with different RE metal ions. It would be preferable in terms of cost to have a Ce or La carboxylate or indeed to be able use a Mischmetal (La, Ce, Pr, and Nd) mixed carboxylate, but some recent inhibitors are best with the smaller Y (above), which is not excessively expensive. Then there is a need for a well-performing and inexpensive carboxylate. There are current efforts to address this problem.

Our proposed model for the formation of a surface protective film involves reaction of the lanthanoid carboxylate with a hydroxidoiron(III) species on the surface to give a mixed iron/rare earth oxido-carboxylate, possibly with Ln-O-Fe bonding [52,57,67].

### 2.5. Rare Earths in Agriculture—Crop Production and Animal Feedstocks

In the 1980s, China embarked on a wide-ranging examination of the effects of rare earths on crop production. Initial results were presented at a conference on “New frontiers in rare earth science and applications” and have appeared as book chapters [68]. In summary, improved crop production was observed for sugar cane, rubber trees, wheat, watermelons, Chinese cabbage, and rice by 5–15% with a good investment-to-profit ratio [69]. Attempts however to reproduce these results under Western conditions have been less successful [70]. Thus, Australian studies were initiated on a number of crops, generally without success, including field trials of a fodder crop in Western Victoria. However, success was reported with sugar cane [70]. The best Chinese results appear to have been achieved with soils having low soluble rare earths, whereas little effect was observed for soils with high soluble rare earths [70]. Earlier Western results were ambivalent and sometimes contradictory, and this was also illustrated at a later (1995) Rare Earth in Agriculture seminar organised by the Australian Academy of Technological Sciences. Following an account of Chinese successes in agriculture [71], the results of a solution culture of corn and mungbean treated with the water-soluble extract of Chinese commercial rare earth fertilisers showed a deleterious effect [72], vividly illustrated, but the pictures are not reproduced in the published proceedings. This work has been published [73,74,75] with a more detailed account of the effects of lanthanum and cerium appearing later [76]. In contrast, pot trials of the effect of lanthanum on barley, canola, and perennial rye grass gave very positive responses [77], also vividly illustrated but not reproduced in the published conference proceedings. This study is detailed in a later publication [78] and report [79]. Three detailed comparisons have been made between Chinese and Western studies of the use of rare earths in agriculture [80,81,82]. The authors of the first review have considerable reservations about the value of rare earth fertilisers owing to Chinese/Western differences, contradictions in Western results (e.g., [72,73,74,75,76,77,78,79]), and a general concern over the control of variables in Chinese studies. However, it seems highly unlikely that the industrial-scale use of rare earth fertilisers in China would be proceeding without a firm cost/benefit analysis. What seems apparent is that low concentrations of rare earths can be beneficial in crop production, but high levels are problematic in increasing production and can lead to toxicity. Even though rare earths can be classified as heavy metals, they have negligible toxicity and clear benefits if appropriate amounts are used. In a similar vein, the heavy metal bismuth has beneficial medicinal properties.

Redling points out that the West took longer to appreciate Chinese work on rare earth feedstock additives [81], but a paper in Chinese appeared in 1991 [83]. It is of interest that Chinese conference presentations of the use of rare earths in agriculture at Australian conferences in 1987 and 1995 did not mention this topic [69,71] and that the several papers (chapters) on agriculture at the groundbreaking conference in China earlier dealt only with plant growth and toxicity [68]. Chinese publications on this topic emerged from 1991 to 2000 and are detailed by Redling [81]. The big incentive for the West to take an interest was the ban on antibiotics as growth enhancers in Switzerland in 1999. Later, this was reinforced by a ban of feed antibiotics in the EU in 2006. These bans have led to a search for alternative growth promoters. European investigations were pioneered by Walter Rambeck at Ludwig-Maximillian University, Munich [84,85], followed by studies in Brunswick, Berlin, and Zurich. Given the centrality of pork in the European diet and the consequent importance of the pig raising industry, it is not surprising that major attention has been directed there. Initial success with piglets [84] was followed by the observation of faster growth and increased size in pigs [85,86,87,88,89]. Similar results were observed in field trials [90]. At the same time, parallel studies showed the low oral toxicity of rare earths [81,91] and negligible uptake into body tissue from field trials with pigs [81,92,93]. As a result of these positive results, the use of rare earth-supplemented feedstocks has been permitted in Switzerland and they were initially marketed as Lancer and Sanocer by Zehentmayer, but currently, Lancer is sold by the major Austrian rare earth company, Treibacher, though Sanocer is still available from Zehentmayer. A veterinary study accompanying the field trials of feeding piglets with feed enriched with 0.25 g/kg of Lancer 500 (La, Ce, Nd, and Pr) showed no enrichment of rare earths in organs, muscle, or fat tissue, representing no danger to consumers. However, somewhat reduced bone strength and bone phosphorus content was observed in the piglets. Significant supplement-induced weight gain was observed [94]. Lancer is now registered for piglets in the EU [95]. There are also reports of a lack of success with the rare earth supplementation of pig feed, and these studies have been detailed [81]. No adverse effects were noted in any of these. In the successful studies, rare earth (mainly La and Ce) citrates perform somewhat better than rare earth chlorides. Possibly, this can be attributed to citrate complexing better to lanthanoids than chloride, and citrate has been used as an eluent for rare earths from ion exchange columns. Better ligation may prevent the diversion of the metals into unprofitable outcomes before the effective targets can be reached.

Investigations have also been extended to poultry, with considerable attention from 2010 onwards [82] and interest in both growth and in egg laying. As with pig production, reports have contradictory features. Thus, a report of increased body weight [96] is offset by two reports of no weight gain [97,98]. Weight gains induced by rare earths were observed for Japanese quails [99,100]. Effects on egg production were also of major interest. A recent study showed that high rare earth supplementation adversely affected the quality of eggs and aspects of metabolism, whereas lower doses increased average egg weight but reduced shell thickness, and there is a need to study whether this can be offset by an adjustment of calcium levels [101]. Several earlier studies reviewed [83] reported increased egg production promoted by rare earths. Effects on ruminants remain relatively uncertain [81,83]. A study of the effects of rare earths on fish growth (carp and rainbow trout) showed no effect on growth and body weight [102], in contrast with Chinese claims.

**Figure 10:**
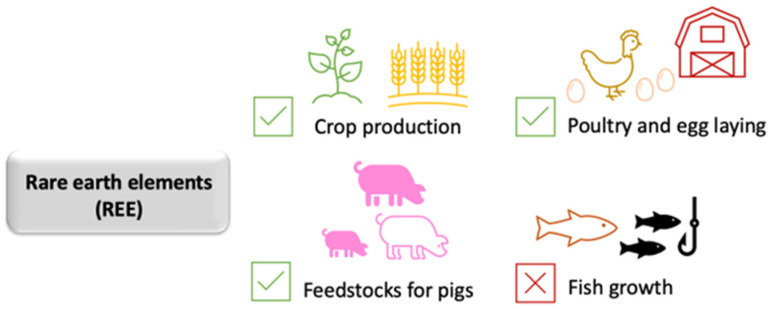
Overview of the animal feedstock and crop rare earth applications. green tick signifies RE enhancement application, red cross signifies RE has no effect overall.

Overall, on balance, it appears that rare earths can be a positive growth enhancer for pigs and poultry but not for fish (Figure 10), and the allowing of commercial use in Switzerland and to a lesser extent in the EU indicate that regulators are satisfied that the practice is without health or environmental risk at the levels where positive growth enhancement has been observed. In a world where increased food production is needed, and where use of antibiotics in growth stimulation should be eliminated, rare earths offer a possible help in the search for food security [103].

### 2.6. Catalysis by Rare Earths

#### 2.6.1. Industrial Catalysts

From the viewpoint of consumption and sustainability, catalytic uses minimise the consumption of rare earths for maximum return. There are three substantial industrial uses of rare earths: (i) in petroleum cracking; (ii) as active supports for exhaust emission catalysts; (iii) in artificial rubber production.

(i) Petroleum Cracking Catalysts

These catalysts (fluid catalytic cracking—FCC) are used to convert various oil feedstocks into more useful products, such as gasoline, diesel, and aviation fuel, as well as propylene as a feedstock for the chemical industry [104]. Zeolite-based catalysts are used, commonly Zeolite Y (not yttrium) and ZSM-5. Rare earths (mainly La and Ce) are added (2–5%) to stabilise the catalyst [104,105,106]. The rare earths offset the dealumination of the zeolite [106], enhance thermal stability, and counter the adverse effects of vanadium [104]. Whilst the amount of rare earths may seem quite low, the huge amounts of FCCs used makes this a major user of La and Ce. As a result of the 2010–2011 rare earth supply crisis, considerable effort was made by catalyst manufacturers to reduce the amount of rare earths in the catalysts because of the then escalating costs, though the price spike did not last. However, catalyst manufacturers have developed alternatives with reduced [107] and even no rare earths [108], though it does not appear that these have taken over [104]. Another issue in the FCC area is the recovery of rare earths from spent catalysts, and a recent paper utilised the reduction of Ce^IV^ to Ce^III^ by peroxide, followed by the extraction of Ce and La by HCl, precipitation with oxalate, and thermolysis to the oxides as a recovery procedure [43]. Another supply crisis would no doubt focus more attention on this source.

(ii) Exhaust Emission Catalysts

Although the ceria-based supports for exhaust emission catalyst are commonly thought of simply as supports, Ce^IV^ in the supports has an active role in the removal of greenhouse gases owing to the oxidising power of cerium in its highest oxidation state [6]. This topic has been comprehensively reviewed recently [6]; hence, a more detailed account is not needed here.

(iii) In Artificial Rubber Production

**Figure 11:**
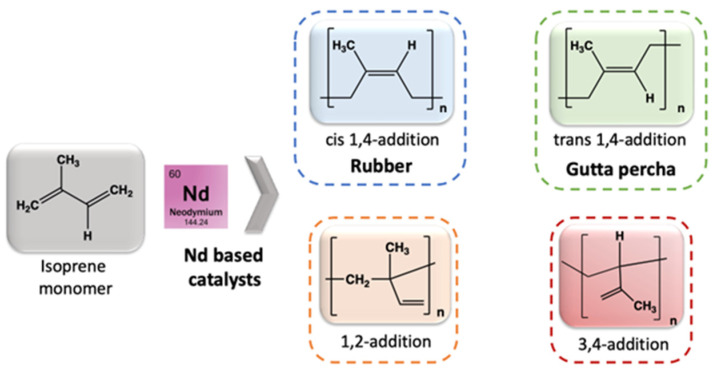
Polyisoprene structures from neodymium-based catalysts.

Since the mid-1980s, Ziegler-Natta-type catalysts incorporating neodymium derivatives have been used in 1,3-diene polymerisation to give poly(1,4-butadienes) for artificial rubber, namely *cis*-1,4-polybutadienes (particularly *cis*-1,4-polyisoprene) (Figure 11) for use in tyres. *Trans*-1,4-polybutadienes give rise to artificial gutta percha (Figure 11), which has more limited use [109]. Binary catalyst systems include a neodymium halide and an aluminium (or Mg) alkyl whilst ternary systems have a neodymium carboxylate, an aluminium alkyl (or Mg), and a halide donor, as the latter increases the *cis*-1,4 specificity [109]. More complex systems are also used, e.g., with the addition of transition metals. Considerable investigation into the neodymium or lanthanoid reagent/aluminium alkyl interactions has been carried out by Anwander [110,111,112,113,114,115]. Whilst it is not possible to detail these studies here, certain highlights should be mentioned. These are establishing Ln(AlR_4_)_3_ species as models for the catalytic behaviour [110,111,112], their capacity to give high *cis*-1,4-polybutadiene specificity [111,112], and the synthesis of a series of mixed carboxylate/tetramethylaluminate complexes Nd(AlMe_4_)_n_(O_2_CR)_3-n_ (n = 0–3) to model the behaviour of Nd carboxylates in catalysis [111]. Understanding the role of chloride donors [111] and modelling *trans*-1,4-polybutadiene formation [114] are other notable achievements. Importantly, evidence suggested that the polymerisation initiator in the neodymium carboxylate system is “Me_2_NdCl” [111]. The contributions and the overall picture have been reviewed [115].

**Scheme 1:**
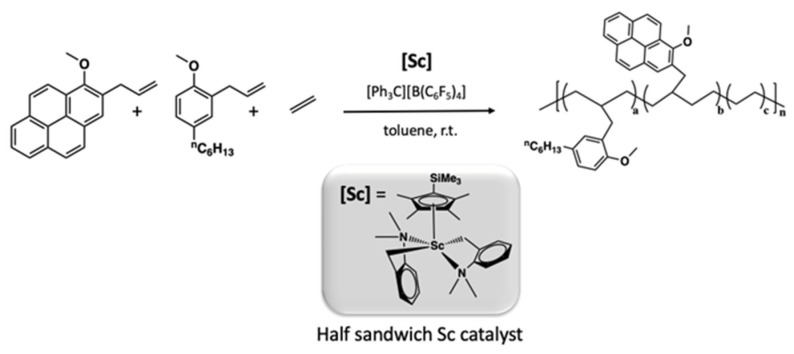
Synthesis of self-healing elastomers using a Sc half sandwich complex.

In addition to the intense research on pre-catalysts for artificial rubber synthesis, many other polymers and co-polymers derived from polar (lactones, lactides, vinylphosphonates, propylene oxide, acrylates, etc.) or non-polar (ethylene, styrene, norbornene, etc.) monomers can be obtained under rare earth catalysis [116,117]. These processes have not yet reached the industrial scale but have been applied to interesting fields, such as in self-healing polymers (Scheme 1) [118]. The most efficient systems for non-polar monomers are based on [(L)LnR_2_]-type complexes, with the rare earth metal bearing one anionic ancillary ligand L (Cp, indenyl, fluorenyl, β-diketonate, etc.) and two R ligands (alkyl, benzyl, and aryl). These complexes are commonly applied in polymerisation reactions after in situ formation of their cationic form via protonation or via borane or carbenium addition.

#### 2.6.2. Lab-Scale Catalysis

**Scheme 2:**
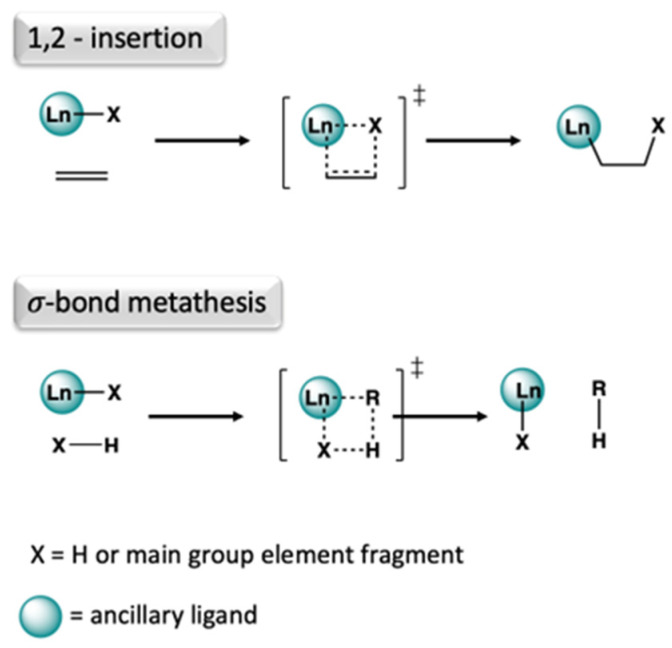
General reactivity pathways characteristic of organolanthanoid complexes.

Rare earth-based inorganic or organometallic complexes have been employed as catalysts or pre-catalysts in organic synthesis and fine chemical synthesis since the 1970s [119]. Rare earth catalysts are interesting (a) for their redox inactivity in the trivalent oxidation state (except Ce, Sm, Eu, and Yb), which allows for reaction fine tuning due to the lanthanide contraction, (b) the Lewis acidity of the metal centre, and (c) the ionic bonding character which provides the possibility of directing regio- and stereo-selective processes only by steric considerations of the ligands and the metal size. In the absence of redox chemistry, the elemental reaction steps in most rare earth-based processes consist in sigma-bond metathesis and 1,2-insertion reactions, as shown in Scheme 2 for hydroelementation reactions (see below). Finally, for the generation of radical species, divalent Sm or tetravalent Ce compounds have found numerous applications, even though these are often under stoichiometric conditions. The advent of photochemistry and electrochemistry has changed this considerably and will further increase the use of these reagents in catalytic processes.

**Scheme 3:**
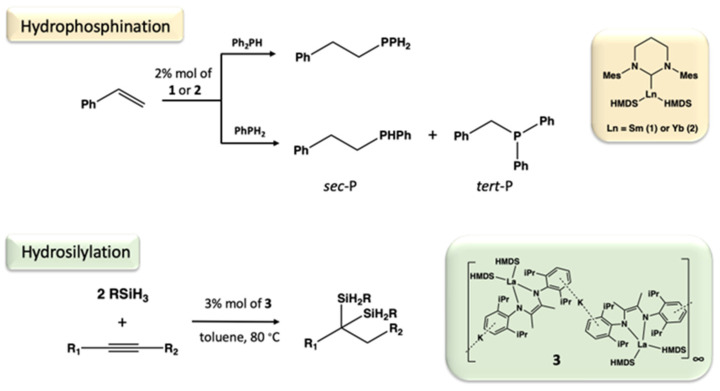
Recent examples of hydroelementation reactions.

Hydroelementation reactions, that is, the addition of an H-E bond (E = H, O, N, P, B, Si) across an unsaturated bond (alkene, alkyne, allene, and carbonyl), have made huge advances thanks to rare earth compounds. The most widely studied reactions are hydroamination processes, which have even led to highly efficient chiral transformations [120,121]. More recently, a wide range of other hydroelementation reactions such as hydrophosphination (Scheme 3) [122], hydrosilylation (Scheme 3) [123], and hydroboration [124,125] have made considerable progress. Furthermore, the cationic rare earth complexes developed initially for their polymerisation activity (see “Industrial catalysis” above) also have high potential for triggering numerous C-H activation and functionalisation processes [126].

**Scheme 4:**
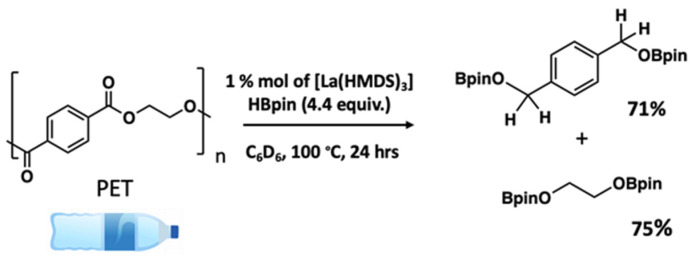
Example of depolymerisation using rare earth metals.

Among the many applications of these processes, an interesting current research direction is the depolymerisation of various polymers [127]. With the growing issue of waste plastic, this will certainly become a major research interest in the coming years. Depolymerisation methods can either provide new organic building blocks or give back the monomers for new polymerisation reactions. For example, Cantat et al. have shown that the reductive hydroboration of polyamides and polyesters using La[HMDS]_3_ (HMDSH = hexamethyldisilazane) is applicable to a wide range of everyday plastic materials, such as PET bottles, stoppers, and CDs, to provide the borylated products (Scheme 4) [128]. Marks et al. used the same cheap and readily available complex for the recovery of ε-caprolactam in high yields from different Nylon sources [129].

**Scheme 5:**
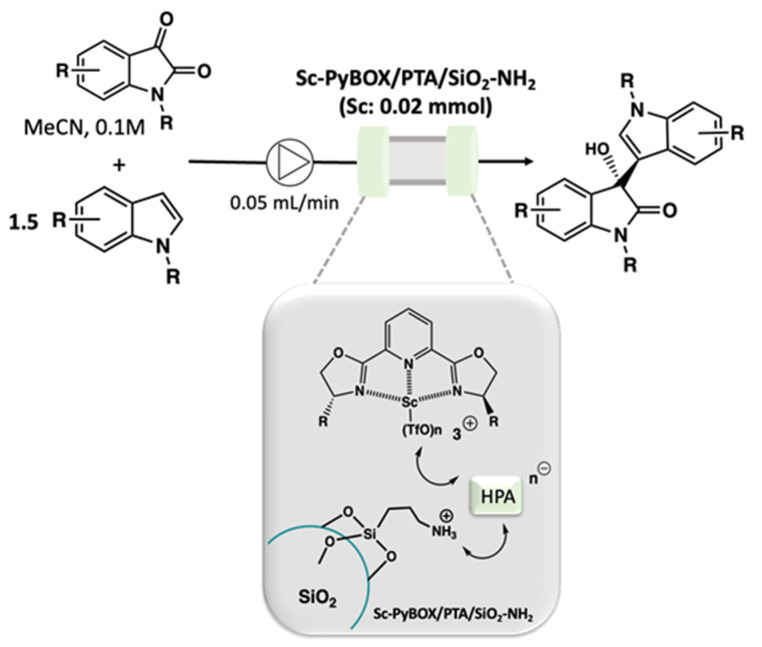
A chiral Sc(OTf)_3_ complex in flow chemistry.

The unique Lewis acidic character of rare earths has led to many interesting transformations in organic synthesis, either alone or in combination with other metals [130,131]. Rare earth triflates, esp. Sc^3+^, La^3+^, and Yb^3+^, are very popular Lewis acids due to their compatibility with water and their easy recovery [130]. The combination with chiral ligands provided many stereoselective processes [132]. Very recently, Kobayashi reported the non-covalent grafting of chiral Sc(OTf)_3_ complexes on inorganic materials, allowing for their implementation in an efficient flow-chemistry process (Scheme 5) [133]. This constitutes a very important step towards future applications, perhaps even on an industrial scale.

**Scheme 6:**
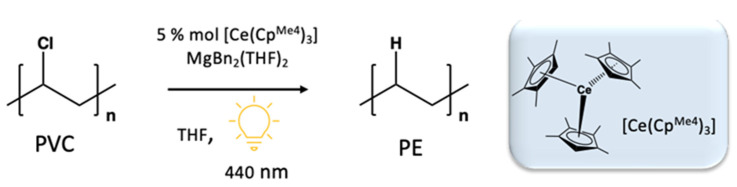
Dechlorination of PVC under photocatalytic conditions and a CeCp_3_ catalyst.

Photocatalysis is a greatly expanding research field in which rare earth complexes are increasingly studied [134,135,136]. In these light-induced processes, rare earths can either act as a photoinitiator, as a photoredox catalyst, or as a (chiral) Lewis acid to trigger the reaction. A great focus has been on the use of Ce^III^/Ce^IV^ chemistry, which has allowed the generation of alkoxy radicals under mild conditions. These underwent a range of further transformations, including HAT reactions on alkanes [137]. Very recently, simple trivalent CeCp_3_ complexes have been applied in photocatalytic dechlorination reactions, including, for example, the dechlorination of PVC (Scheme 6) [138]. With the emergence of new Ln^4+^ chemistry, this area holds promise for further important discoveries. It should further be mentioned that a breakthrough has now also been achieved using divalent europium in catalysis. The divalent europium complexes bearing a coumarin or carbostyril chromophore in the ligand can be generated photocatalytically and applied in many reduction or coupling reactions [139].

**Scheme 7:**
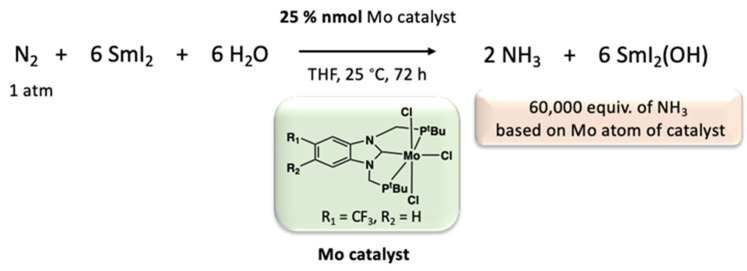
Mo-catalysed ammonia production using SmI_2_ as a stoichiometric reductant.

Synthetic organic electrochemistry is another important domain where rare earth metals will certainly contribute significantly in the future [140,141]. As mentioned above, many radical reactions based on stoichiometric or super stoichiometric amounts of SmI_2_ have seen the light of day [142]; however, rendering them catalytic is still highly challenging [143]. Several electrochemical processes to access divalent Sm have been reported [140,141]. The invention by Mellah et al. of using Sm electrodes for sacrificial and non-sacrificial processes has certainly been a milestone in this field [144,145]. With these different electro-catalytic methods, the use of chiral ligands in SmI_2_ chemistry may become foreseeable and may further increase the synthetic potential of this already-rich chemistry. An example of high interest for the catalytic regeneration of SmI_2_ is the molybdenum-catalysed production of ammonia from water or alcohols and nitrogen gas developed by Nishibayashi (Scheme 7) [146]. This outstanding process, providing up to 230,000 eq. of ammonia per Mo complex, has several advantages over the high energy-consuming Haber–Bosch process as it works at room temperature using water or alcohols as readily available hydrogen sources. Nevertheless, to date, stoichiometric amounts of SmI_2_ are required.

### 2.7. Health

**Figure 12:**
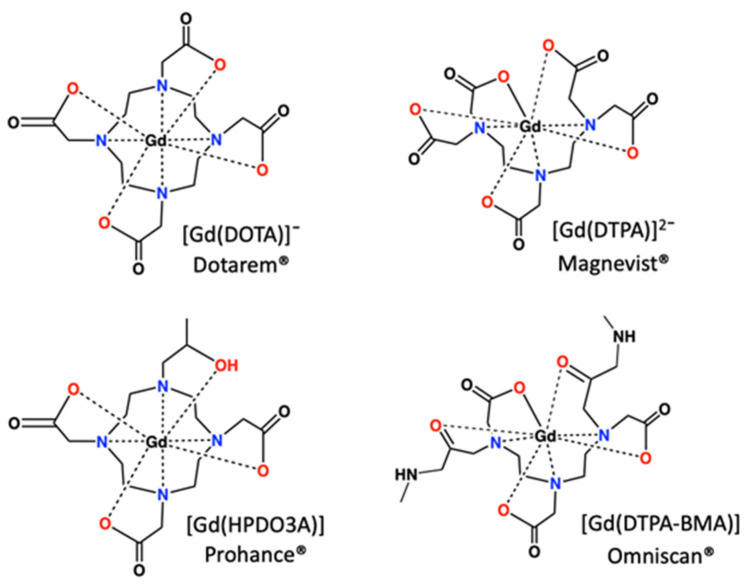
Commercially available medical imaging agents.

At first sight, a connection between rare earths and health may seem remote, since they are commonly held to have no essential biological role [70,147], but microorganisms requiring a lanthanoid to function have now been found [148,149,150]. In addition, the role that they have in agriculture indicates they can have a significant contribution (Section 2.5). However, gadolinium magnetic imaging has been known for many years [151,152,153]. Examples of medical imaging agents currently utilised are shown in Figure 12. Limited examples of adverse reactions to these agents are known to be usually associated with severe renal impairment [154]. Their capacity to replace Ca^2+^ in biological systems is not only well known but has been exploited in studying Ca^2+^ metabolism [147]. The size similarity between appropriate RE^3+^ and Ca^2+^ and the higher charge of the former favours replacement, and the spectroscopic and luminescent properties of Ln^3+^ then provide a detection handle that is not possible for the spectroscopically silent Ca^2+^ [155].

The considerable importance of rare earths in health prompted Chemical Society Reviews to publish, an editorial and a series of reviews on this topic in 2006 [151,155,156,157]. In his review of the therapeutic uses of lanthanoids, Fricker points out that many early prospective therapeutic uses of rare earths have come to nought [155]. In the health area as in agriculture (above), low doses may be beneficial but high doses are not. Two lanthanoid-based drugs that continue to be in use are Flammacerium and Fosrenol. The former is useful in the treatment of burns and contains cerium nitrate as well as silver sulfadiazine. The cerium salt is included for antibacterial properties and is of particular value where more sophisticated forms of treatment are not available. Fosrenol has lanthanum carbonate as the active ingredient and is used to lower phosphate levels in end-stage renal disease patients being treated by dialysis [155]. A possible role of GdCl_3_ in mitigating toxicant-induced liver damage has been indicated [155], and recent studies indicate continuing interest in this area (e.g., [158,159]), but the treatment is a long way from medical use at present. Recently, the modification of luminescent lanthanoid complexes in order for them to be used in biomedical applications has been reviewed [160].

Besides the use of gadolinium complexes in MRI, there are several actual and prospective uses of lanthanoid complexes and nanoparticles in the detection and treatment of cancer [149,152,153]. Indeed, there are so many that only a limited account can be provided here. Lanthanoid complexes of the FDA-approved porphyrin-based drug, Photofrin, show promise for photodynamic therapy [153], but further development is needed. Nanoparticle-based analogues have also been produced. The attachment of lanthanoid radioisotopes to antibodies or already approved anticancer drugs has proven to be a way of delivering targeted radiation [153]. Obviously a very desirable outcome is to combine the detection/mapping of tumours and treatment in a single system, for example, the use of mixtures of metal oxide nanoparticles to perform both functions or preparation of a single-phase nanoparticle of a mixed metal oxide [161]. Mixed metal oxides have the advantage over mixed metal complexes of a higher load of the requisite metals. This much to be desired combination of imaging and treatment is termed Cancer Theranostics, and it has been the subject of three substantial recent reviews [162,163,164]. The former review considers in detail the radiochemical advantages and disadvantages of a whole range of accessible isotopes to provide a basis for the choice of imaging and scanning options. Particularly, the authors cover the route for the relevant isotope production and the isotopic properties. A highly desirable situation is where different isotopes of the same element can perform different functions, as this simplifies the chemistry and biochemistry involved [162]. The second is focused more on clinical outcomes and rare earth compounds are considered only as part of a much wider range of radiochemical reagents [163]. The third covers the more specialized area of lanthanoid porphyrinoids as molecular theranostics [164]. The overall aim of theranostics is to provide personalised medicine tailored to individual needs, but this requires ready access to a very complete range of isotopes as well as the appropriate carrier molecules.

**Figure 13:**
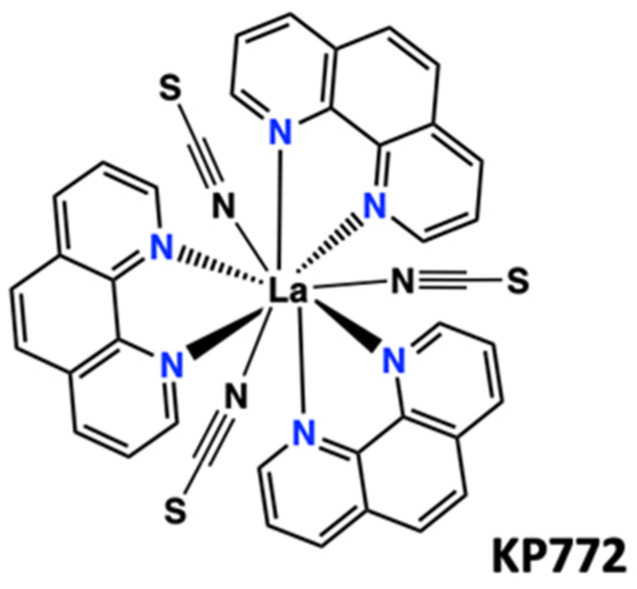
Chem draw representation of drug-resistant cancer complex KP772.

Rare earths have also featured in the search for new chemotherapy agents with some emphasis on derivatives of lanthanum [165]. A complex of particular interest is *tris*-1,10-phenanthrolinetri-(*iso*thiocyanato)lanthanum(III) (KP772) (Figure 13), which has the capacity to overcome drug resistance [166]. Two types of photoactive phenanthroline or polypyridyl lanthanoid complexes have also been reported around that time [167,168]. More recently, KP772 was shown to overcome multiple drug resistance in leukaemia and lymphoma cancer cells with the apoptotic effect in resistant cells being superior to that in parent cells [169]. The mechanisms of apoptosis and resistance were determined. KP772 appears to warrant considerable further study. A current report describes the antitumour activity of [LaCl_2_(phen)_2_(μ-Cl)]_2_ (phen = 1,10-phenanthroline) but appears unaware of KP772 [170], as are other recent studies of antitumour 1,10-phenanthroline-lanthanoid(III) complexes [171,172]. A number of other lanthanoid cytotoxic agents have been reviewed [153]. An interesting class are lanthanoid complexes of the 5,7-dibromo- and 5,7-dichloro-8-quinolinolate ligands, where the cytotoxicity is enhanced from those of the active proligands [173]. These results are also considered against a background of the anticancer activity of other metal 8-quinolinolates [174]. A completely new approach to chemotherapy has recently been reported in which a metallofullerenol, Gd@C_82_(OH)_22_, was used to encage rather than kill a tumour [175]. It will be interesting to see how this method develops. Overall, there appears to be promise of a clinical lanthanoid antitumour agent, but clinical testing is now needed.

### 2.8. Material Science

As already mentioned in different sections of this review, bulk materials containing rare earths are omnipresent in everyday life now, especially as phosphors, magnets and sensors in lighting, and in electronics and energy applications. This section provides an overview on some recent developments of rare earth-based materials, including molecular entities (single molecules, fullerenes, and nanorings) as well as more extended systems (nanoparticles, nanowires, MOFs, coordination polymers, thin films, and glasses). A wide-spread panel of applications have been proposed with these materials, which could lead to important advances in the areas of data management (storage, encryption, and reading), quantum computing, electronics, telecommunication, switches, biology, medicine, displays, gas storage and detection, anti-counterfeiting, and many more.

#### 2.8.1. Luminescence

The unique light-emitting properties of Ln^3+^ ions (sharp and well-defined emission bands) have been studied for a long time and led to numerous applications [176,177]. Due to the weak emission of Ln^3+^ ions (forbidden f-f transitions), many applications require organic light-harvesting ligands attached or in close proximity to the lanthanoid ion for metal sensitization (antenna effect) [178] and a small selection of some remarkable recent results are presented.

In a recent study, the anticancer agent doxorubicin was employed to sensitize the NIR emitter Yb^3+^. The incorporation of doxorubicin and amphiphilic Yb^3+^ chelates into liposomes allowed for the direct monitoring of drug release: the sensitized emission of Yb^3+^ was shown to be dependent on the integrity of the particles. This could even be evidenced in experiments with living mice [179].

**Scheme 8:**
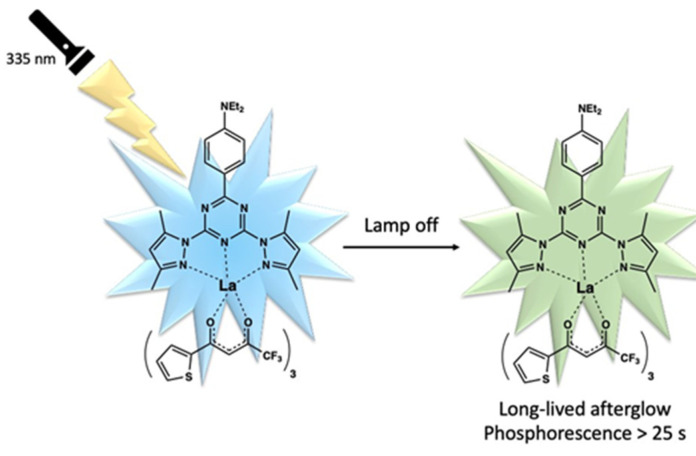
Ultralong phosphorescence in an organolanthanoid complex.

The importance of the ligand environment in light-emitting complexes was shown in the charging and ultralong phosphorescence of a lanthanoid-facilitated organic complex (Scheme 8). In the long-lived phosphorescent lanthanoid compound, green luminescence was observed for up to 30 s under cryogenic conditions (77 K) using laser excitation [180].

The real-time monitoring of a chemical reaction in physiological environments with high specificity (bioorthogonal reactions) is highly challenging. Very recently, a Eu^III^-based complex was reported as a small-molecule optical imaging agent which showed off–on luminescence and enabled the quantitative analysis of the progress of the bioorthogonal reaction. The characteristic signal was achieved through efficient energy harvesting and transference to the Eu^III^ from the expansion of the conjugated system of the antenna [181].

The combination of crystals of two distinct light-emitting Tb and Dy complexes via soft-crystal polymerization between Tb^III^ and Dy^III^ coordination centres on the crystal surfaces was achieved, leading to the long-range energy transfer from Dy^III^ to Tb^III^ in the crystal (ca. 150 µm) of a photonic molecular train [182].

**Scheme 9:**
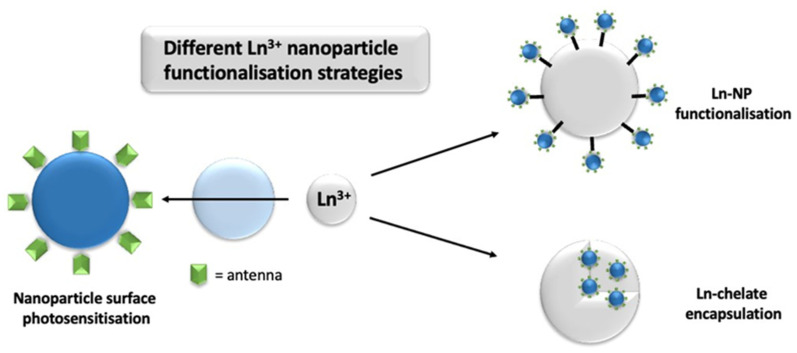
Different Ln-F NPs.

The matrix around the Ln ion also plays a crucial role for luminescence applications. The combination of lanthanoids with dyes and quantum dots is increasingly used in Time-Gated Förster Resonance Energy Transfer TG FRET for multiplexed biosensing and bioimaging. Applications are found in immunoassays, RNA/DNA assays, optical barcoding, and in vivo or in vitro imaging [183]. Small amounts of Ln ions can be incorporated into nanostructured inorganic materials. This lanthanoid ion doping can lead to numerous applications involving luminescence as recently reviewed [184]. Ln-fluoride nanoparticles (NPs) are widely used tools, as fluorides have low phonon energy (≈350 cm^−1^) and high chemical stability [185,186]; hence, intense luminescence can be obtained. Such Ln-F NPs are especially applied in upconversion processes (UC), i.e., the conversion of two or more low-energy photons into one high-energy photon. The combination of dye (sensitizer) and the Ln ion can be placed at different sites of the NP (Scheme 9). Whereas most UC NPs involve lanthanoid ions, recently, the first d-f upconversion was reported with a Ru-Yb supramolecular assembly [187]. Furthermore, sensitization in a discrete Er complex provided highly efficient NIR to visible light upconversion [188]. Among many applications, UC NPs have been used in anti-counterfeiting and forensics [189,190].

An interesting application of downconversion was reported in plant growth experiments by the use of transparent films equipped with a Eu^3+^ complex as a UV-to-red wavelength-converting luminophore. These films absorb UV light and exhibit strong red luminescence under sunlight, leading to significant growth acceleration with size increment and biomass production for vegetable crops and trees [191], a greener approach than rare earth fertilisers (Section 2.5) but still at an early stage.

Lanthanoid-based nanoscintillators are increasingly studied, as they exhibit X-ray excited long persistent luminescence [192]. An application was recently shown in a swallowable X-ray dosimeter for the real-time monitoring of radiotherapy [193]. Another area with a spectacular growth over the last 15 years is the field of Ln-based thermometers as non-contact thermal probes [194]. The unique properties of Ln ions (versatility, stability, and narrow emission band profiles of the ions that cover the entire electromagnetic spectrum with relatively high emission quantum yields) have provided many applications, e.g., as new tools for thermal imaging or early tumour detection. Ratiometric lanthanoid-based luminescent thermometers are increasingly studied with a variety of Ln-based materials, including MOFs. In a very recent example, crosslinking through uncoordinated −NH_2_ or COOH on Tb-MOFs reacting with the epoxy groups on triglycidyl isocyanurate (TGIC) provided a highly sensitive thermometer in different temperature ranges after curing [195]. A nice example of a dual magneto-optical molecular thermometer based on an air-stable Dy^3+^ complex was reported recently which combines high-performance SMM and Boltzmann-type luminescence thermometry [196]. It was observed that the synergy between multiparametric magneto-optical readouts and multiple linear regression afforded a 10-fold improvement in the relative thermal sensitivity of the thermometer over the whole temperature range.

Another interesting aspect of lanthanoid luminescence has recently emerged in chiral lanthanoid complexes. These complexes can serve as chiral probes for circularly polarized luminescence (CPL) [197,198,199]. More recently, the use of helicene-based ligands around Ln centres has led to the observation of CPL and magnetochiral dichroism, even at room temperature [200]. Potential applications of such complexes are in the fields of optical data readout and display technology.

A recent study has reinvestigated molecular Eu^3+^ crystals as a new platform for photonic quantum technologies [201]. Optical fibres play a highly important role in telecommunications. In order to increase the signal intensity of data transmissions, erbium-doped fibre amplifiers are used. They consist of fused silica fibres doped with Er_2_O_3_. The efficient emission of Er^3+^ at 1550 nm improves data transmission in the *C*-band (1530 and 1565 nm) [202,203]. It was shown that tapered optical fibres coated with rare earth complexes could find quantum applications [204]. Lanthanoids also have a fundamental role in lasers. Accordingly, nonlinear optical (NLO) crystals have been used to magnify the laser frequency and show great relevance in laser chemistry. Significant interest surrounds rare earth compounds having flexible coordination geometries leading to distorted structural motifs providing non-centrosymmetric compounds that contribute to beneficial second harmonic generation [205].

Lanthanoid ion-doped glasses or glass ceramics are considered as a novel generation of luminescence materials and potentially have many applications in optical fibres, solid-state lasers, optical thermometry sensors, waveguides, infrared detectors, and display devices. Two recent reviews show the progress of lanthanoid-doped fluorosilicate glasses [206] and of Dy^3+^-doped glasses for white light emission [207].

#### 2.8.2. Magnetism

The paramagnetism of lanthanoid ions was long associated with NMR shift reagents. More recently, enormous progress has been observed in single-molecule magnets (SMMs) based on lanthanoid ions, and especially Dy^3+^ [208,209,210,211,212,213,214].

**Figure 14:**
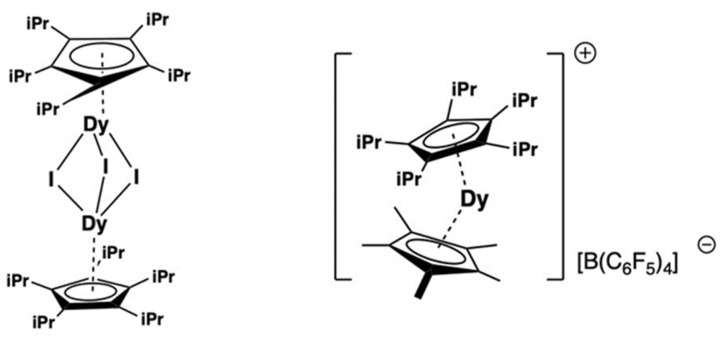
High-performance Dy-based SMMs [211].

Figure 14 gives an overview of the most powerful SMMs reported to date, showing magnetic behaviour up to 80 K and broad hysteresis with an inversion barrier U_eff_ of up to 1800 cm^−1^. It should be noted that in addition to metallocene-type complexes and other metal–organic complexes, SMM behaviour was also observed in fullerenes [215,216]. A recent example of magneto-electric coupling shows the high potential for possible applications in data management [217].

#### 2.8.3. Redox

The last 25 years have witnessed an enormous development in the redox chemistry of lanthanoids. Molecular compounds in the divalent state, long-time restricted to Sm, Eu, and Yb, have now been reported for all the Ln series, except the radioactive Pm [218].

In the tetravalent state, Pr^4+^ and Tb^4+^ have joined Ce^4+^ molecular complexes [219,220]. The combination of Ln ions with redox-active ligands has even allowed for access to a formal mono-valent Ce complex [221]. All these insights open up the way to new applications, especially in the field of redox switches [222]. Examples are the redox luminescence switching in nanowires [223] and the temperature-controlled redox isomerism in a bimetallic Yb^3+^ complex bearing a redox active bis(aryl)acenaphthenequinonediimine ligand [224]. Furthermore, a study in 2019 revealed the possibility of using unusual divalent complexes as redox qbits [225].

#### 2.8.4. Various Applications

The field of molecular machines has seen considerable growth over the last 25 years [226]. Even though many metals have been studied, examples involving rare earth metals are still rare [227]. In an important study, a network of porphyrin-based Eu double-decker complexes was bound onto a Cu surface and showed simultaneous and coordinated rotational switching when applying an electric field from the tip of a scanning tunnelling microscope [228].

Ln oxide thin films are studied in a large variety of electronic processes. Such films are mainly produced via ALD or MOCVD using organometallic or metal-organic precursors [116,229].

Ln-MOFs have been shown to provide excellent properties for gas storage and separation, e.g., with MFM-300(Sc) [230]. MOFs can further find numerous applications in host–guest chemistry, as drug carriers, in cancer diagnosis and therapy, and in fluorescence sensing or catalysis [230,231,232,233,234]. A very recent example shows that multimetallic Ln-MOFs (Eu, Yb, and Gd) have high potential as optical tags through orthogonal luminescence lifetime encoding [235].

## 3. Conclusions

The extravagant title for this review can be justified by the widespread role rare earths have in daily life, even if the public is largely unaware of them. As they have recently been classified as “critical metals”, they may be entering public consciousness more. Their most immediate interaction with the public is with the use of iron neodymium boride magnets in cars, especially in electric vehicles, and in computers, but cerium-based ceramics have long been used as exhaust emission catalyst supports and indeed can have an active catalyst role themselves. In these roles alone, rare earths make a significant contribution to environmental improvement and to sustainability, and the magnets have an important role in green wind power electricity. Rare earth ores are widely available, especially in Australia, but supplies of separated rare earths are constrained by the Chinese domination of this market. However, alternatives are becoming available with Lynas Rare Earths approaching supply of 10% of the demand and with plans for new separation plants in the USA. Separation methods as well as recycling procedures often are far from green with the use of phosphate or organophosphorus ester solvent extractants in kerosene, but alternatives are being developed with particular interest in magnet recycling because of the amount of material available and becoming available, and catalysts supports are a potential bulk source of cerium. Although fluid cracking catalysts (FCCs) have only a low % of rare earths, the total large amounts in use suggest potential as a rare earth source. Of course, their use as exhaust emission supports and in FCCs is likely to decline with a reduction in fossil fuel usage, and their availability as a rare earth source will rise at least for some years as a result.

With the corrosion of steel and other structures being a massive problem and the need to replace toxic chromate corrosion inhibitors, rare earth inhibitors and particularly rare earth carboxylates have emerged as a greener alternative, one which would lead to a new bulk use of rare earths. The use by China of rare earths to enhance crop production for 40 years is a testimony to their lack of toxicity, even if the increases in crop yields have not been replicated in the West. It should be noted that the results were often obtained on soils with low rare earth content. However, their use to enhance growth in pigs and chickens has been well demonstrated through the work of Walter Rambeck of the Animal Physiology Department of LMU Germany, and they are licenced for this purpose in Switzerland. In the EU, where use of antibiotics for growth enhancement has been banned, [Lancer^R^], mainly cerium citrate, is permitted as a food additive for young piglets. What is evident with use of rare earths is that low dosages are effective, but higher levels may induce toxicity. Rare earth catalysts are used industrially in petroleum cracking, exhaust emission catalysts, and catalysts for artificial rubber production. They have a wide range of uses as lab-scale catalysts, some of which may attain industrial applications in due course, for example, depolymerisation catalysts. Uses in the health arena are widespread and growing. Gadolinium-based imaging is well established, as is the use of lanthanoid isotopes, in the detection and treatment of cancer, and it is only a matter of time before a chemotherapeutic drug reaches the clinical trial phase. Applications in the materials area are dominated by those based on luminescence where there is overlap with health applications. Films produced by MOCVD and ALD have wide applications, and single-molecule magnets are approaching the point where real-life applications will emerge.
